# Development of a loop-mediated Isothermal amplification assay for sensitive and rapid detection of *Vibrio parahaemolyticus*

**DOI:** 10.1186/1471-2180-8-163

**Published:** 2008-09-30

**Authors:** Wataru Yamazaki, Masanori Ishibashi, Ryuji Kawahara, Kiyoshi Inoue

**Affiliations:** 1 Division of Bacteriology, Osaka Prefectural Institute of Public Health, Osaka, Japan

## Abstract

**Background:**

*Vibrio parahaemolyticus *is a marine seafood-borne pathogen causing gastrointestinal disorders in humans. Thermostable direct hemolysin (TDH) and TDH-related hemolysin (TRH) are known as major virulence determinants of *V. parahaemolyticus*. Most *V. parahaemolyticus *isolates from the environment do not produce TDH or TRH. Total *V. parahaemolyticus *has been used as an indicator for control of seafood contamination toward prevention of infection. Detection of total *V. parahaemolyticus *using conventional culture- and biochemical-based assays is time-consuming and laborious, requiring more than three days. Thus, we developed a novel and highly specific loop-mediated isothermal amplification (LAMP) assay for the sensitive and rapid detection of *Vibrio parahaemolyticus*.

**Results:**

The assay provided markedly more sensitive and rapid detection of *V. parahaemolyticus *strains than conventional biochemical and PCR assays. The assay correctly identified 143 *V. parahaemolyticus *strains, but did not detect 33 non-*parahaemolyticus Vibrio *and 56 non-*Vibrio *strains. Sensitivity of the LAMP assay for direct detection of *V. parahaemolyticus *in pure cultures and in spiked shrimp samples was 5.3 × 10^2 ^CFU per ml/g (2.0 CFU per reaction). The sensitivity of the LAMP assay was 10-fold more sensitive than that of the conventional PCR assay. The LAMP assay was markedly faster, requiring for amplification 13–22 min in a single colony on TCBS agar from each of 143 *V. parahaemolyticus *strains and less than 35 min in spiked shrimp samples. The LAMP assay for detection of *V. parahaemolyticus *required less than 40 min in a single colony on thiosulfate citrate bile salt sucrose (TCBS) agar and 60 min in spiked shrimp samples from the beginning of DNA extraction to final determination.

**Conclusion:**

The LAMP assay is a sensitive, rapid and simple tool for the detection of *V. parahaemolyticus *and will facilitate the surveillance for control of contamination of *V. parahaemolyticus *in seafood.

## Background

*Vibrio parahaemolyticus *is a marine seafoodborne pathogen causing gastrointestinal disorders in humans [1,2]. Thermostable direct hemolysin (TDH) and TDH-related hemolysin (TRH) are known as major virulence determinants of *V*.*parahaemolyticus *[3]. This bacterium is widely present in estuarine, marine, and coastal environments throughout the world [1,2]. Therefore, ingestion of raw or undercooked seafood contaminated with *V*.*parahaemolyticus *is risk factors in humans [1,2].

Most *V*.*parahaemolyticus *isolates from the environment do not produce TDH or TRH. Virulent strains of *V. parahaemolyticus *are usually found together with larger populations of avirulent strains in the environment [1,2,4,5]. The similarity in growth kinetics of the virulent and avirulent strains is a major obstacle for selective detection of virulent strains in seafood. Total *V. parahaemolyticus *has thus been used as an indicator for control of food contamination toward prevention of infection. Thermolabile hemolysin (*tlh*) has been characterised by Taniguchi and colleagues [6], which has been found in all *V. parahaemolyticus *isolates. [4-7]. This hemolysin is species specific and not a virulence factor. This gene is therefore useful target for detection of total *V. parahaemolyticus*.

Detection of total *V. parahaemolyticus *using conventional culture- and biochemical-based assays is time-consuming and laborious, requiring more than three days. A rapid, reliable and practical assay for the detection of total *V. parahaemolyticus *has been sought. Several PCR assays offer a more sophisticated approach to the identification of *V. parahaemolyticus *[4,8]. Although PCR assays provide more rapid identification of *V. parahaemolyticus *than conventional biochemical-based assays, they require electrophoresis in an agarose gel, which is time-consuming and tedious. Real time PCR assays recently developed for identification of *V. parahaemolyticus *[5,7] are rapider than conventional PCR assays due to the detection of fluorescence from amplification. Real-time PCR assay is, however, not routinely used due to the requirement for an expensive thermal cycler with a fluorescence detector.

Among other techniques one promising candidate is a novel nucleic acid amplification method termed loop-mediated isothermal amplification (LAMP) [9-11]. Several investigators have developed LAMP assays for detection of pathogenic microorganisims [12-17]. LAMP assay is faster and easier to perform than conventional PCR assays, as well as being more specific [14,15]. Furthermore, because the LAMP assay synthesizes a large amount of DNA, the products can be detected by simple turbidity. Thus, compared to PCR assays, expensive equipment is not necessary to give a high level of precision [12,14,15]. These features allow simple, rapid and cost-effective detection [15,16]. Also, the increase in the turbidity of the reaction mixture according to the production of precipitate correlates with the amount of DNA synthesized [13-15]. In addition, the preparation steps of the LAMP assay are fewer than with conventional PCR and real-time PCR assays, and LAMP assays require less time than those assays [18]. Although various LAMP assays for the identification of pathogenic organisms have been developed, no assay for the detection of *V. parahaemolyticus *has been described.

Here, we describe a sensitive, rapid and simple LAMP assay for the detection of *V. parahaemolyticus*. Sensitivity was determined in pure cultures and in spiked shrimp samples.

## Results

**Figure 1:**
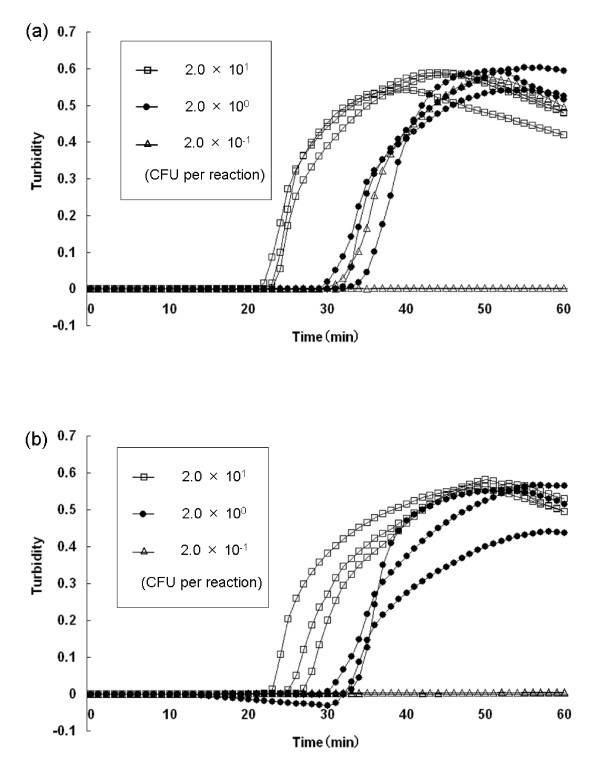
**Sensitivity test for detection of *V. parahaemolyticus *in pure cultures and in spiked shrimp samples by real-time turbidimetry**. The curves from left to right indicate decreasing concentrations of CFU from bacterial colonies [2.0^1 ^to 2.0^-1 ^CFU per reaction]. (a) Detection of *V. parahaemolyticus *in pure cultures; (b) detection of *V. parahaemolyticus *in spiked shrimp samples.

LAMP products were detected from all 143 *V. parahaemolyticus *strains. No LAMP products were detected from any of the 33 non-*parahaemolyticus Vibrio *and 56 non-*Vibrio *strains (Table 1). The PCR assay required more than 4 h, while the LAMP assay was markedly faster, requiring for amplification 13–22 min in a single colony on TCBS agar from each of 143 *V. parahaemolyticus *strains and less than 35 min in spiked shrimp samples (Fig. 1). The assay required less than 40 min and 60 min for detection of *V. parahaemolyticus *in a colony on TCBS agar and in spiked shrimp samples from the beginning of DNA extraction to final determination.

**Table 1:** Results of the LAMP assay for detection of *V. parahaemolyticus*

Species	No. of strains tested	Positive number by LAMP
*V. parahaemolyticus*	143	143
*V. fluvialis*	10	0
*V. vulnificus*	10	0
*V. cholerae*	5	0
*V. alginolyticus*	2	0
*V. furnissii*	2	0
*V. mimicus*	2	0
*V. harveyi*	1	0
*V. metschnikovii*	1	0
*Grimontia hollisae*	5	0
Other bacteria^a)^	51	0

^a)^ Described in the Methods section.

As shown in Table 2, sensitivities of the LAMP assay for *V. parahaemolyticus *AQ4980 in pure cultures and in spiked shrimp samples were found to be 5.3 × 10^2 ^CFU per ml (2.0 CFU per reaction). Further, the sensitivity of the LAMP assay was 10-fold higher than that of the PCR assay (Table 2). The dilutions yielding 19.6 -2.0 CFU per reaction showed an increase in turbidity (Fig. 1) and was visible as white turbidity but not that of 0.2 CFU per reaction. Sensitivities determined by the two methods were constantly matched with each other. When LAMP assay performed in triplicate using 1 μl templates DNA containing 1.0 CFU per reaction from pure cultures, two of three samples showed positive results, as well as using 2 μl templates DNA from shrimp samples (Data not shown).

**Table 2:** Sensitivity of the LAMP assay for *V. parahaemolyticus*

Strain	Samples		Dilutions of cultures for the assays				
			
			10^-1^	10^-2^	10^-3^	10^-4^	10^-5^
*V. parahaemolyticus*							
AQ4980							
	Pure cultures	CFU per reaction	196.3	19.6	2.0	0.2	0.02
		LAMP	+	+	+	± (1/3)	-
		PCR	+	+	± (1/3)	-	-
							
	Spiked shrimps	CFU per reaction	196.3	19.6	2.0	0.2	0.02
		LAMP	+	+	+	-	-
		CFU per reaction	19.6	2.0	0.2	0.02	0.002
		PCR	+	-	-	-	ND

+, triplicate assay showed all positive.

±, triplicate assay showed both positive and negative (positive number/tested number).

-, triplicate assay showed all negative.

ND, Not determined.

## Discussion

The bacterial culture test for the isolation and identification of *V. parahaemolyticus *from food samples after enrichment requires 2–3 d, with plating onto selective agars, sequential subculture and biochemical characteristic test. In contrast, the LAMP assay was markedly faster. Conventional PCR assay requires 4–5 h for amplification, electrophoresis and staining, while the LAMP assay requires for DNA extraction from samples and amplification less than 40–60 min. Further, amplification of the LAMP assay could be judged by visual assessment using the naked eye, without the need for electrophoresis. The LAMP assay was more sensitive, rapid and simple than the conventional PCR assay. Therefore, the LAMP assay is more effective in detecting *V. parahaemolyticus *than the conventional PCR assay.

In the preliminary tests, the LAMP assay using 1, 2 and 4 μl templates DNA from shrimp samples was enough for amplification without any inhibition of the reaction in 25 μl reaction mixture volumes. The PCR assay was, however, shown inhibited results in 50 μl reaction mixture volumes. No PCR products were detected from the PCR reaction mixtures containing 4 μl templates from shrimp samples. Slight bands were detected from the mixtures containing 2 μl templates. Clear bands were obtained from each of the mixture containing 1 μl or 0.4 μl templates. It is known that some components of food inhibit DNA amplification such as LAMP and PCR assays [5,15]. The results seem to suggest LAMP is more resistant than PCR assay to inhibition by shrimp components.

Contamination of *V. parahaemolyticus *in seafood is one of the most important public health hazards [1,2,4,5,7]. The frequent outbreaks caused by *V. parahaemolyticus *worldwide highlight the need for control of contamination of *V. parahaemolyticus *in seafood. Especially, ingestion of fresh raw seafood as sushi and sashimi dishes in Japan is a risk factor to humans [19]. The total number of *V. parahaemolyticus *cells in fish fillets and shellfishes is required below 100 CFU/g for raw consumption by law in Japan. To reduce the risk of *V. parahaemolyticus *infection and to ensure food safety, our LAMP assay would be significant in the detection of total *V. parahaemolyticus *in routine tests and in potentially applying to the most-provable-number method. Rapid, simple and sensitive assay for detection of *V. parahaemolyticus *by LAMP should facilitate the surveillance for control of contamination of *V. parahaemolyticus *in seafood. The LAMP assay has potential value for rapid and simple screening of total *V. parahaemolyticus*-contaminated samples before they are consumed. Although the shrimp samples were artificially spiked, we successfully developed the first LAMP assay for detection of total *V. parahaemolyticus *from seafood samples. Development of the LAMP assay for detection of virulent strains of *V. parahaemolyticus *is required, as well as real-time and conventional PCR assays [4,5,7,8]. Further studies will be performed for detection of virulent *V. parahaemolyticus *and evaluation of the assay using a large number of seafood naturally contaminated by *V. parahaemolyticus*.

## Conclusion

The LAMP assay provided markedly more sensitive, simple and rapid detection of *V. parahaemolyticus *than conventional biochemical and PCR assays. The LAMP assay for detection of *V. parahaemolyticus *required less than 40 min in a colony on TCBS agar and 60 min in spiked shrimp samples from the beginning of DNA extraction to final determination. The LAMP assay is a powerful tool for the rapid and sensitive detection of *V. parahaemolyticus*, and will facilitate the surveillance for control of contamination of *V. parahaemolyticus *in seafood.

## Methods

### Bacterial strains

A total of 232 bacterial strains were used, including 143 *Vibrio parahaemolyticus *strains and 89 non-*V. parahaemolyticus *strains. One-hundred twenty one *V. parahaemolyticus *clinical strains were obtained from clinical stricken overseas travelers and domestic cases between 1986 and 2008 in Japan. Twenty-two *V. parahaemolyticus *strains were obtained from seafood samples purchased at supermarkets in Osaka prefecture, Japan, 2008. Seventeen non-*V. parahaemolyticus *reference strains were obtained from international culture collections (*Arcobacter butzleri *ATCC 49616^T ^(American Type Culture Collection, USA); *Arcobacter cryaerophilus *ATCC 43158^T^; *Arcobacter skirrowii *ATCC 51132^T^; *Campylobacter coli *JCM 2529^T ^(Japan Collection of Microorganisms, Saitama, Japan); *Campylobacter fetus *subsp. *fetus *ATCC 27374^T^; *Campylobacter jejuni *subsp. *jejuni *ATCC 33291, ATCC33292; *Campylobacter lari *JCM 2530^T^; *Campylobacter upsaliensis *ATCC 43954^T^; *Escherichia coli *ATCC 25922, and ATCC 35218; *Pseudomonas aeruginosa *ATCC 27853; *Staphylococcus aureus *subsp. *aureus *ATCC 25923;*Staphylococcus epidermidis *ATCC 12228;*Vibrio alginolyticus *IFO 15630^T ^(Institute for Fermentation, Osaka, Japan); *Vibrio harveyi *IFO 15634^T^; and *Vibrio vulnificus *IFO 15645^T^). A superscript T designates a type-strain. Thirty non-*parahaemolyticus Vibrio *strains were obtained from clinical patients, food or environmental sources, as follows: 10 *V. fluvialis*; 9 *V. vulnificus*; 5 *V. cholerae*; two strains each of *V. furnissii *and *V. mimicus*; and one strain each of *V. alginolyticus*, and *V. metschnikovii*. Forty-two non-*Vibrio *strains were obtained from clinical patients, food or environmental sources, as follows: seven heat-labile enterotoxin (LT)-producing *Escherichia coli*; five *Grimontia hollisae*; five LT non-producing *Escherichia coli*; and one strain each of *Acinetobacter baumannii*, *Aeromonas hydrophila*, *Aeromonas sobria*, *Citrobacter freundii*, *Enterobacter cloacae*, *Enterococcus faecalis*, *Enterococcus faecium*, *Enterococcus gallinarum*, *Haemophilus influenzae*, *Helicobacter pylori*, *Klebsiella oxytoca*, *Klebsiella pneumoniae*, *Morganella morganii*, *Plesiomonas shigelloides*, *Proteus mirabilis*, *Providensia alcalifaciens*, *Pseudomonas putida*, *Salmonella enterica *serovar Enteritidis, *Shigella flexneri *1a, *Serratia marcescence*, *Shigella sonnei*, *Staphylococcus captis*, *Streptococcus agalactiae*, *Streptococcus pneumoniae*, and *Streptococcus pyogenes*.

### Storage and culture conditions

All *Vibrio *and *G. hollisae *strains were stored in 4 ml of marine semi-solid broth (4.8 g agar No.1, Oxoid; 1 L seawater), Casitone semi-solid broth (Eiken Chemical Co., Ltd., Tokyo, Japan) or cooked meat broth (Becton Dickinson and Co., Sparks, MD, USA) at room temperature until required. They were grown on thiosulfate citrate bile salt sucrose agar (TCBS agar; Eiken Chemical) or tryptic soy agar (TSA; Nissui, Tokyo, Japan) supplemented with 2.5% (w/v) NaCl and incubated overnight at 35–37°C. Storage and culture conditions of other bacterial strains were followed as previously described [14].

### DNA extraction from culture

Bacterial DNA was extracted as previously described [14]. In brief, a single loopful of culture on TCBS agar, TSA supplemented with 2.5% (w/v) NaCl or blood agar was inoculated in 50 μl of NaOH (25 mM) in a 1.5-ml microcentrifuge tube using a disposable loop (1-mm diameter), and the cell mixture was heated at 95°C for 5 min. After neutralization with 4 μl of Tris-HCl buffer (1 M), cell debris was pelleted by centrifugation at 20,000 *g*, 4°C, for 5 min and the supernatant was used as template DNA for the LAMP assay.

### LAMP assay

LAMP assay was performed as previously described [14]. The final LAMP assay comprised 2 μl of template DNA, 1 μl of *Bst *DNA Polymerase (Eiken Chemical) and each of LAMP primers in a 1 × Reaction Mix (Eiken Chemical). Final volume was adjusted to 25 μl. All primers were designed from sequence data submitted to GenBank (thermolabile hemolysin gene, *tlh*, M36437) [6] The sequences and locations of each primer are shown in Table 3. The concentrations and production steps of LAMP primers, and the amplification conditions were followed as previously described [14]. The reaction was considered to be positive when the turbidity reached 0.1 within 60 min using a Loopamp real-time turbidimeter (LA-320; Teramecs, Kyoto, Japan). Turbidity visible with the naked eye was also considered to indicate a successful LAMP procedure.

**Table 3:** LAMP primers used

Primer	Sequence	Gene location (bp)
Tlh-FIP	ATG TTT TTA AAT GAA ACG GAG CTC CGG CAA AAA ACG AAG ATG GT (F1c-F2)	392-368 (F1c), 321–339 (F2)
Tlh-BIP	ACG TCG CAA AAC GTT ATC CGG CGA AGA ACG TAA TGT CTG (B1-B2c)	406–425 (B1), 487-467 (B2c)
Tlh-F3	AGC TAC TCG AAA GAT GAT CC (F3)	283–302
Tlh-B3	GGT TGT ATG AGA AGC GAT TG (B3c)	511-492
Tlh-LF	ACC AGT AGC CGT CAA TG (LFc)	367-351
Tlh-LB	TTA GAT TTG GCG AAC GAG A (LB)	445–463

All primers were designed from the sequence of *tlh *gene of *V. parahaemolyticus *M36437, submitted to GenBank by Taniguchi *et al*., 1986 [6].

### Determination of sensitivity of the LAMP assay in pure cultures

The sensitivity of the LAMP assay for the detection of *V. parahaemolyticus *in pure cultures was determined as previously described [14] with slight modification using known amounts of *V. parahaemolyticus *AQ4980. In brief, small number of cells from a single culture on TCBS agar was inoculated in 4 ml of tryptic soy broth (TSB; Becton Dickinson) and incubated overnight at 35°C. Then, 40 μl of enriched TSB was transferred to a new 4 ml of TSB and incubated 4 h with shaking at 150 rpm at 35°C to obtain mid-log phase cells. Serial 10-fold dilutions of the cultures were prepared in PBS (Phosphate-buffered saline). For preparation of DNAs from pure cultures, 100 μl of each was transferred to a 1.5-ml microcentrifuge tube, and was centrifuged for 5 min at 20,000 *g*. After removal of the supernatant, the pellets were resuspended in 50 μl of NaOH (25 mM), and the mixture was heated at 95°C, for 5 min. After neutralization with 4 μl of Tris-HCl buffer (1 M, pH 7.5), debris was pelleted by centrifugation at 20,000 *g*, 4°C, for 5 min. Two microliters of each supernatant was then used as template DNA for LAMP assay. The sensitivity tests of the LAMP assay were conducted in triplicate, and the detection limits were defined as the last positive dilutions, with the sample considered positive if all three samples tested positive. In parallel, to enumerate the bacteria, 100-μl aliquots of appropriate dilutions were spread on TSA supplemented with 2.5% NaCl in duplicate and incubated overnight at 37°C. Colonies were counted at the dilution yielding 30 to 300 Colony Forming Units (CFUs), and CFU per ml of suspension was calculated.

### Determination of sensitivity of the LAMP assay in shrimp samples

Shrimp samples were purchased at a supermarket in Osaka, Japan, 2007. The shrimp samples were determined to be negative for *V. parahaemolyticus *according to the results of a microbiological examination with overnight APW (Alkaline Peptone Water; Eiken Chemical) enrichments and subsequent plating onto TCBS agar. Two-hundred twenty-five millilitres of APW were added to 25 g of the shrimp sample, which was then homogenized by a stomacker (Pro-media, SH-001; ELMEX Ltd., Tokyo, Japan) for 30 s. Serial 10-fold dilutions of mid-log phase *V. parahaemolyticus *cells were prepared as described above. One-hundred microlitters of each mid-log phase *V. parahaemolyticus *cells was spiked into 900 μl of each of the shrimp homogenates. After mixing well, each homogenate was centrifuged at 900 *g *for 1 min to remove larger debris. The supernatant was transferred to a new 1.5-ml microcentrifuge tube, and was centrifuged for 5 min at 10,000 *g*. After removal of the supernatant, the pellets were resuspended in 100 μl of NaOH (25 mM), and the mixture was heated at 95°C, for 5 min. After neutralization with 8 μl of Tris-HCl buffer (1 M, pH 7.5), debris was pelleted by centrifugation at 20,000 *g*, 4°C, for 5 min. Four microliters of each supernatant was then used as template DNA for LAMP assay. The sensitivity tests of the LAMP assay were conducted in triplicate, and the sensitivity of the LAMP assay was determined as described above.

### PCR assay

A PCR assay targeting the *tlh *gene was performed in a 50-μl reaction mixture containing 2 μl of template DNA from pure cultures or 0.4 μl of that from spiked shrimp samples, PCR buffer, 1 unit of TaKaRa ExTaq Polymerase (TaKaRa Bio Inc., Shiga, Japan), 4 μl of dNTP mixture (2.5 mM, TaKaRa Bio), and the respective primer (Hokkaido System Science) in 1 × ExTaq buffer (TaKaRa Bio). The sequences of primers were as described in a published paper [4]. The concentrations of both primers were adjusted 0.2 μM. DNA amplification was performed in a TaKaRa PCR Thermal Cycler Dice Gradient (TaKaRa Bio). The cycling conditions used were one cycle of 94°C for 3 min, 35 cycles each of 94°C for 1 min, 55°C for 1 min and 72°C for 1 min, and ending with a final extension time at 72°C for 5 min. Samples were held at 4°C prior to analysis. PCR products were subjected to electrophoresis in 2% agarose gels. After staining with ethidium bromide, the PCR products were detected under UV light. The sensitivity of the PCR assay was determined using template DNA from pure cultures and spiked cells in shrimp sample as described above. The sensitivity tests of the PCR assays were conducted in triplicate, and the detection limits were defined as the last positive dilutions, with the sample considered positive if all three samples tested positive.

## Authors' contributions

WY carried out LAMP and PCR assays; WY and MI conceived the study. WY, RK and MI isolated and identified bacterial strains together; KI coordinated the study. All authors read and approved the final manuscript.
